# The impact of simulated 3x3 tournament on vertical jump force-time metrics in national team male basketball players

**DOI:** 10.3389/fphys.2024.1447343

**Published:** 2024-09-11

**Authors:** Dimitrije Cabarkapa, Jelena Aleksic, Darko Krsman, Damjana V. Cabarkapa, Nicolas M. Philipp, Andrew C. Fry

**Affiliations:** ^1^ Jayhawk Athletic Performance Laboratory – Wu Tsai Human Performance Alliance, Department of Health, Sport and Exercise Sciences, University of Kansas, Lawrence, KS, United States; ^2^ Faculty of Sport and Physical Education, University of Belgrade, Belgrade, Serbia; ^3^ International Strength and Conditioning Institute, Novi Sad, Serbia

**Keywords:** force, power, concentric, eccentric, athlete monitoring, biomechanics, sport

## Abstract

With innovative portable force plate systems being widely implemented for lower-body neuromuscular performance assessment in an applied sports setting and the existing gap in the scientific literature regarding player performance during in-game competitive scenarios, the purpose of the present study was to compare changes in countermovement vertical jump (CVJ) performance pre-post a simulated 3×3 basketball tournament. Seven current or former members of a 3×3 national basketball team volunteered to participate in the present investigation. Upon completing standardized warm-up procedures, athletes stepped on a uni-axial force plate system sampling at 1,000 Hz and performed three maximal-effort CVJs with no arm swing. Then, the athletes proceeded to play a simulated 3×3 basketball tournament composed of two consecutive games, separated by a 15-min rest interval. Immediately following the completion of the second game, the identical CVJ testing procedures were repeated. Paired sample t-tests were used to examine pre-post-tournament differences in nineteen CVJ performance metrics (*p* < 0.05). The results reveal that force-time metrics during both eccentric and concentric phases of the CVJ remain relatively unchanged pre-post simulated 3×3 basketball tournament. However, multiple force-time metrics within the eccentric phase of the CVJ changed by 12.1%–19.1% (e.g., eccentric peak power and peak velocity, eccentric duration), suggesting that the eccentric phase of CVJ might be responsive to performance stimulus to a greater extent than the concentric phase. Overall, these findings further support the importance of comprehensive CVJ analysis when intending to measure changes in neuromuscular performance.

## 1 Introduction

Over the past decade, three-on-three (3×3) basketball has gained global recognition, including its inclusion as an official sport in the 2020 Tokyo Olympics (Cabarkapa et al., 2023a). The game features several rule adjustments compared to the standard five-on-five (5×5) competitive format such as a reduced number of players (i.e., three starters and one reserve), smaller court dimensions (i.e., 15 m × 11 m), and 10-min match duration with a 12-s shot clock (Cabarkapa et al., 2023a; Conte et al., 2019; Csurilla et al., 2023). In addition, 3×3 basketball is played with a single hoop without breaks after scoring, which increases the intensity of the gameplay and fosters continuous scoring opportunities (Conte et al., 2019).

While 3×3 basketball is a relatively recent research topic of interest within the area of sports science, the data about this domain of athlete performance is rapidly expanding (Cabarkapa et al., 2023a; Conte et al., 2019; Montgomery and Maloney, 2018). The majority of research reports have been focused on comparing physiological and physical demands between the 3×3 and 5×5 competitive formats (Cabarkapa et al., 2024; Conte et al., 2019; Figueira et al., 2022; Leite et al., 2013; McGown et al., 2020; Willberg et al., 2022). These studies consistently highlight the significantly higher workloads experienced by 3×3 players, as they tend to spend more time playing at near maximum intensities, surpassing 90% of their maximal heart rate (Cabarkapa et al., 2023a; Leite et al., 2013; McGown et al., 2020). In addition, Conte et al. (Conte et al., 2019) brought attention to remarkably shorter durations of live and stoppage time phases in 3×3 basketball games (i.e., approximately <20 s), with a ratio between these two phases being 0.92 ± 0.13. This work-to-rest ratio surpasses the one observed within the traditional 5×5 competitive format, which typically ranges from 1:2 or higher (Bender, 2019).

Despite the previously mentioned literature comparing the physiological and physical demands of these two styles of basketball play, a significant research gap still exists in understanding player performance characteristics at the elite level of 3×3 competition, especially during live game scenarios (McGown et al., 2020; McCormick et al., 2012). This scarcity of data can be attributed to the inherent challenges posed by competition and sports club regulations, where testing procedures on actual game days may potentially disrupt the team’s performance (Scanlan et al., 2012). Thus, such constraints make it difficult to gain a thorough understanding of the physical and physiological demands of the competition as well as how they relate to the player’s performance (Willberg et al., 2022).

To address these challenges, researchers are increasingly turning to field-based simulation tests as a means to replicate the demands of actual competition (Scanlan et al., 2012; Scanlan et al., 2018). In this context, portable force plates are often employed to collect force-time data in applied sports settings (Cabarkapa et al., 2023b; Philipp et al., 2023a). The countermovement vertical jump (CVJ) serves as a common testing modality used to evaluate players’ neuromuscular performance in a non-invasive and time-efficient manner (Philipp et al., 2023a; Cabarkapa et al., 2023a; Merrigan et al., 2022). This assessment method is especially valuable in sport-specific environments, offering direct on-field insight into players' performance capacities (Claudino et al., 2017). In the context of basketball, CVJ assessment has been used to differentiate between players included in the starting line-up and their substitutions (Cabarkapa et al., 2023d), detect position-specific differences (Cabarkapa et al., 2023e), and difference players across various competitive levels (Pehar et al., 2017). Also, CVJ assessment has been used to monitor fatigue-induced changes in neuromuscular performance pre-post practice (Cabarkapa et al., 2023c), as well as over the course of an entire basketball season (Philipp et al., 2023a). However, there is a scarcity of literature employing in-depth CVJ assessment to distinguish changes in force-time metrics pre-post an actual competition, especially within the top-tier 3×3 basketball athletes.

Therefore, with innovative portable force plate systems being widely implemented for lower-body neuromuscular performance assessment in an applied sports setting and the existing gap in the scientific literature regarding player performance during in-game competitive scenarios, the purpose of the present study was to compare changes in some of the most commonly examined CVJ force-time metric pre-post simulated 3×3 basketball tournament.

## 2 Materials and methods

### 2.1 Participants

Seven basketball players (age = 19.2 ± 1.1 years; height = 193.3 ± 7.2 cm; body mass = 84.6 ± 9.5 kg) volunteered to participate in this study. The players were current or former members of a 3×3 national basketball team. All athletes were free of musculoskeletal injuries and actively participated in individual and/or team strength and conditioning and basketball-specific training activities at the time point of the data collection. The testing procedures performed in this investigation were previously approved by the University of Kansas Institutional Review Board (No. 00149094) and all participants signed an informed consent document.

### 2.2 Procedures

Upon arrival at the outdoor basketball court that corresponds to 3×3 official regulations, all athletes completed a standardized warm-up procedure (10–15 min) composed of dynamic stretching exercises (e.g., A-skips, butt-kicks, high knees, side-to-side lunges, high-knee-pulls) administered by a Certified Strength and Conditioning Specialist. Following familiarization with the testing procedures, all athletes stepped on a dual uni-axial force plate (ForceDecks Max, VALD Performance, Brisbane, Australia) sampling at 1,000 Hz and performed three maximal-effort CVJs with no arm swing (i.e., hands on the hips during the entire movement). Each jump trial was separated by a 10–15 s rest interval and the mean value across three trials was used for performance analysis purposes. Strong verbal encouragement was provided throughout testing procedures and the athletes were instructed to focus on pushing against the ground as explosively as possible (Kershner et al., 2019).

After completion of initial CVJ testing procedures, the participants proceeded with playing a simulated 3×3 basketball tournament composed of two games, separated by a 15-min rest interval. The games were played according to 3×3 basketball rules (i.e., no break after a scored basket, 12-s shot clock, no half-time or quarters). The winner of the game was the team that first reached the 21-point score or the team that had more points at the 10-min mark. The gameplay was only paused when the free-throw shots were attempted (i.e., 10–15 s). To ensure that athletes were competing to the best of their ability, members of the coaching staff were present to observe the simulated tournament. Immediately following the completion of the second game, the identical CVJ testing procedures were repeated (i.e., pre-post simulated tournament). In addition, all athletes shared approximately the same amount of playing time (i.e., administered by the coach’s game plan). The testing procedures were conducted on an official 3×3 outdoor basketball court between 17:00–19:00h on a sunny day (21°C) with moderate humidity (43%).

### 2.3 Variables

The dependent variables examined in the present study were: braking phase duration, eccentric braking impulse, concentric impulse, eccentric and concentric duration and peak velocity, peak and mean force and power during both eccentric and concentric phases of the CVJ movement, contraction time, jump height (i.e., impulse-momentum calculation), reactive strength index (RSI)-modified (i.e., jump height divided by contraction time), and countermovement depth. The selection of the force-time metrics of interest, automatically computed via VALD performance analysis software that demonstrated adequate levels of validity and reliability, was based on previously published research reports (Cabarkapa et al., 2023a; Merrigan et al., 2022; Cabarkapa et al., 2023b; Anicic et al., 2023; Merrigan et al., 2024; Philipp et al., 2023b; Philipp et al., 2023c). Additional information pertaining to data analysis software can be found at https://valdperformance.com/forcedecks/.

### 2.4 Statistical analysis

Shapiro-Wilk test corroborated that the assumption of normality was not violated. Descriptive statistics, means and standard deviations, were calculated for each dependent variable analyzed in the present study. Paired sample t-tests were used to examine pre- to post-tournament differences in each CVJ force-time metric of interest. The percent difference was calculated for each dependent variable. Due to the small sample size (n = 7), Hedges’ *g* was calculated to determine effect size (*g* = 0.2 – small effect, *g* = 0.5 – moderate effect, *g* = 0.8 – large effect) (Hedges, 1981; Cabarkapa et al., 2022). All statistical analyses were completed with SPSS (Version 26.0; IBM Corp., Armonk, NY, USA).

## 3 Results

Descriptive statistics for each dependent variable examined in this study are presented in Table 1. Besides the difference in eccentric mean force that was trivial in magnitude (*g* = 0.052), no statistically significant differences were observed in any CVJ force-time metrics of interest. The percentage difference pre-post-tournament across all dependent variables ranged between 0.7% and 19.1%. In addition, the majority of the effect sizes were small to moderate (*g* = 0.052–0.556) in magnitude, except breaking phase duration, eccentric duration, and contraction time, which demonstrated large effect size differences (*g* = 0.835–0.906).

**TABLE 1:** Descriptive data (mean ± standard deviation) and comparison statistics, including effect size (ES) and percentage difference (%-diff), for changes in each dependent variable examined pre- to post-tournament.

Variable [unit]	Pre-tournament	Post-tournament	*p*-value	ES	%-diff
*Eccentric phase*					
Braking phase duration [s]	0.296 ± 0.078	0.246 ± 0.033	0.139	0.835	18.5
Eccentric braking impulse [N·s]	53.7 ± 17.4	60.8 ± 4.8	0.224	0.556	12.4
Eccentric duration [s]	0.531 ± 0.099	0.461 ± 0.046	0.183	0.906	14.1
Eccentric peak velocity [m·s^-1^]	−1.14 ± 0.33	−1.29 ± 0.28	0.144	0.490	12.3
Eccentric peak force [N]	2114.1 ± 280.9	2220.4 ± 244.3	0.331	0.404	4.9
Eccentric mean force [N]	834.3 ± 94.6	829.4 ± 92.4	0.011	0.052	0.6
Eccentric peak power [W]	1,414.4 ± 669.3	1712.3 ± 609.8	0.183	0.465	19.1
Eccentric mean power [W]	471.0 ± 171.1	512.4 ± 162.9	0.222	0.247	8.4
*Concentric phase*					
Concentric duration [s]	0.240 ± 0.036	0.222 ± 0.031	0.060	0.536	7.8
Concentric impulse [N·s]	0.249 ± 0.025	0.245 ± 0.028	0.387	0.151	1.6
Concentric peak velocity [m·s^-1^]	3.03 ± 0.13	3.01 ± 0.21	0.533	0.114	0.7
Concentric peak force [N]	2257.2 ± 253.1	2353.9 ± 189.2	0.218	0.432	4.2
Concentric mean force [N]	1885.3 ± 172.6	1944.6 ± 161.4	0.189	0.354	3.1
Concentric peak power [W]	5314.7 ± 511.5	5369.1 ± 609.1	0.670	0.097	1.0
Concentric mean power [W]	2992.9 ± 285.3	3071.7 ± 328.8	0.491	0.256	2.6
*Other*					
Contraction time [s]	0.771 ± 0.127	0.683 ± 0.053	0.147	0.904	12.1
Jump height [cm]	44.4 ± 4.0	43.5 ± 6.3	0.541	0.171	2.0
RSI-modified [ratio]	0.60 ± 0.10	0.64 ± 0.10	0.519	0.400	6.5
Countermovement depth [cm]	−29.5 ± 9.2	−27.9 ± 6.7	0.209	0.198	5.6

Note: RSI, reactive strength index.

## 4 Discussion

To the best of our knowledge, this is the first study that examined pre-post changes in neuromuscular performance characteristics of top-tier 3×3 basketball players. The results reveal that force-time metrics during both eccentric and concentric phases of the CVJ remain relatively unchanged. No statistically significant differences were observed in any variables of interest except eccentric mean force, which was trivial in magnitude (*g* = 0.052). However, multiple metrics such as eccentric braking impulse, eccentric duration, braking phase duration, and eccentric peak velocity and power did demonstrate moderate to large effect size magnitudes (*g* = 0.456–0.906). Overall, these data suggest that the eccentric phase of CVJ tends to be affected by performance stimulus to a greater extent than the concentric phase.

Previous research focused on examining the acute impact of fatigue on neuromuscular performance characteristics has obtained mixed findings (Cabarkapa et al., 2023c; Cabarkapa D. V. et al., 2023; Gathercole et al., 2015; Hoffman et al., 2002; Spiteri et al., 2013). Some research reports have revealed a notable decrease in multiple force-time metrics (Cabarkapa et al., 2023c; Spiteri et al., 2013), while others observed no changes in CVJ performance parameters during practice as well as an official competition (Cabarkapa D. V. et al., 2023; Gathercole et al., 2015; Hoffman et al., 2002). A recently published study by Cabarkapa et al. (Cabarkapa et al., 2023c) found a significant decrease in concentric impulse, peak velocity, and mean and power pre-post practice when examining a cohort of professional 5×5 basketball players, which is contradictory to the results obtained in the present investigation. The observed discrepancy in the findings may be attributed to the sport-specific demands as well as the level of play (e.g., first and second-tier professional basketball league vs. national team). Unlike the 5×5 competitive style that requires a considerable aerobic energy contribution (Ostojic et al., 2006), a 3×3 basketball game largely relies on anaerobic energy demands (Cabarkapa et al., 2023a; Leite et al., 2013; McGown et al., 2020). The game is played for 10 min on a smaller court and without breaks after the basket is scored. Although the intensity of gameplay within that short timeframe might be higher (Cabarkapa et al., 2023a; Leite et al., 2013; McGown et al., 2020), the overall workloads placed on 3×3 basketball players during practice or competition might be lower when compared to their 5×5 counterparts and be incapable of inducing a considerable reduction in CVJ performance. Still, it should be noted that superior aerobic fitness may allow 3×3 athletes to play the game at a faster pace as it can provide a solid base that meets the on-court performance demands (e.g., low efficiency of aerobic energy system may limit athlete’s competitive ability) (Ostojic et al., 2006). Also, considering that the participants examined in this study were top-tier athletes selected to play for the national team, we can assume that the inability to detect significant changes in neuromuscular performance may be attributed to the fact that these athletes were adequately trained to sustain on-court competitive demands imposed by a simulated 3×3 basketball tournament.

Despite no statistically significant differences in CVJ performance metrics being observed in all dependent variables of interest examined in the present study, except eccentric mean force which was trivial in magnitude, it should be noted that multiple metrics did demonstrate moderate to large effect sizes. Eccentric peak power and peak velocity experienced a moderate increase (19.1% and 12.3%, respectively), while eccentric duration and overall contraction time were reduced (14.1% and 12.1%, respectively). Considering that no change in eccentric mean and peak force was observed pre-post simulated 3×3 basketball tournament, we can conclude that this increase in eccentric peak power was primarily driven by an increase in the velocity of the movement (i.e., *power= force x velocity*) (Fry et al., 2019). Based on unpublished data from our laboratory, these results seem to follow the same trend observed within the CVJ data collected on professional cohorts of team-sport athletes (e.g., handball, basketball, volleyball), where exercise-induced fatigue tends to have a greater impact on the eccentric phase of the CVJ than the concentric phase. In a similar manner, a slight decrease observed in the overall CVJ contraction time seems to be mainly induced by a decrease in the eccentric phase duration, as pre-post tournament change in concentric duration was notably shorter. Lastly, the results of this study reveal that jump height as one of the most commonly used performance measures tends to be resistant to detecting fatigue-induced performance changes (i.e., 2.0% pre-post-tournament change). This further supports previously published research by Merrigan et al. (Merrigan et al., 2020) that indicates that being solely focused on examining outcome metrics such as jump height may fail to provide in-depth insight pertaining to movement strategy (e.g., how the specific outcome was achieved). Lastly, while further research is warranted on this topic, it should be noted that some of the observed positive changes in force-time metrics could be influenced by a potentiation effect, given that previous research has found that the potentiation effect does not dissipate 5–6 min post-exercise completion (Chiu et al., 2023; Philipp et al., 2023b; MacIntosh et al., 2012).

While the findings of the present study provide additional insight into neuromuscular performance characteristics of top-tier 3×3 basketball players and how they change in response to a simulated tournament play, this study is not without limitations. As with many investigations conducted on a cohort of professional athletes, one of the limitations is the sample size, which could have been larger. Another limitation relates to the inability to quantify the external load (i.e., the amount of work performed) that the examined group of 3×3 athletes was exposed to. This information could provide a better understanding of factors that contributed to changes in CVJ force-time metrics and it presents one of the future research directions. Moreover, additional research is warranted to examine if these findings are sex-specific, if they can be applied to other 3×3 basketball competitive levels (e.g., junior, semi-professional), and if they are impacted by athletes’ recovery strategies (e.g., sleep, supplementation).

## 5 Conclusion

In conclusion, the results of the present investigation reveal that force-time metrics during both eccentric and concentric phases of the CVJ remain relatively unchanged pre-post simulated 3×3 basketball tournament. However, it should be noted that multiple force time metrics within the eccentric phase of the CVJ changed by 12.1%–19.1% (e.g., eccentric peak power and peak velocity, eccentric duration), suggesting that the eccentric phase of CVJ might be responsive to performance stimulus to a greater extent than the concentric phase. These findings may be used by sports practitioners when assessing athletes’ readiness to compete, monitoring fatigue-recovery neuromuscular performance changes, and ability to sustain game-like exercise stimulus. Moreover, these findings further support the importance of comprehensive CVJ analysis when intending to measure changes in neuromuscular performance.

## Data Availability

The raw data supporting the conclusions of this article will be made available by the authors, without undue reservation.
